# Sons al Balcó: A Comparative Analysis of WASN-Based *L_Aeq_* Measured Values with Perceptual Questionnaires in Barcelona during the COVID-19 Lockdown

**DOI:** 10.3390/s24051650

**Published:** 2024-03-03

**Authors:** Daniel Bonet-Solà, Pau Bergadà, Enric Dorca, Carme Martínez-Suquía, Rosa Ma Alsina-Pagès

**Affiliations:** HER—Human-Environment Research, La Salle-Universitat Ramon Llull, Sant Joan de la Salle, 42, 08022 Barcelona, Spain; daniel.bonet@students.salle.url.edu (D.B.-S.);

**Keywords:** lockdown, soundscape, *L_Aeq_*, annoyance, perception, WASN, Barcelona

## Abstract

The mobility and activity restrictions imposed in Spain due to the COVID-19 pandemic caused a significant improvement in the urban noise pollution that could be objectively measured in those cities with acoustic sensor networks deployed. This significant change in the urban soundscapes was also perceived by citizens who positively appraised this new acoustic scenario. In this work, authors present a comparative analysis between different noise indices provided by 70 sound sensors deployed in Barcelona, both during and before the lockdown, and the results of a perceptual test conducted in the framework of the project *Sons al Balcó* during the lockdown, which received more than one hundred contributions in Barcelona alone. The analysis has been performed by clustering the objective and subjective data according to the predominant noise sources in the location of the sensors and differentiating road traffic in heavy, moderate and low-traffic areas. The study brings out strong alignments between a decline in noise indices, acoustic satisfaction improvement and changes in the predominant noise sources, supporting the idea that objective calibrated data can be useful to make a qualitative approximation to the subjective perception of urban soundscapes when further information is not available.

## 1. Introduction

The lockdown period during the COVID-19 outbreak at the beginning of 2020 allowed a new possibility not recorded yet: to be able to dismiss the human effect on many situations and confront unexplored scenarios, which could give clues to better understand human behavior. One of these situations was the reduction in acoustic noise level in urban and suburban areas since many people were confined at home or at least were subjected to severe mobility restrictions.

The lockdown gave the scientific community a unique scenario to assess how the reduction in noise levels affects the human perception of the acoustic environment. It is expected that the lower noise levels measured during the confinement translate to higher levels of acoustic satisfaction by the population. However, noise levels alone do not explain the complexity of the subjective acoustic comfort appraisal. In fact, the type of predominant noise source should be taken into account [1]. Therefore, a combined analysis of objective noise indices and the subjective assessment of the soundscapes performed during the lockdown, taking into account the predominant noise sources present in different areas, could give a very useful insight into which regulations should have a greater impact on acoustic comfort (e.g., reducing traffic density in quieter residential areas or limiting night-time leisure activities). As these kind of regulations normally face a strong rejection by part of the citizenship (such as private vehicle owners or people related to the leisure sector), assessing and comparing their expected effectiveness beforehand is essential.

In a previous research, ref. [2] data gathered from acoustic sensors scattered in diverse acoustic points in a medium-sized city (i.e., Girona, Spain) were compared with the answers reported in a poll and conducted around the same points. The authors were aware of the very small set of subjective data analyzed. However, it satisfied the goal of opening the door to the possibility of evaluating the soundscape by means of both objective and calibrated measurements and perceived appraisal by citizens. The answers reported in the survey, despite being a preliminary analysis with limited data, were able to distinguish the sound sources present around a certain sensor and match those noise sources detected in sensors data analysis. The results presented in that work also aligned with the objective measurements related to noise levels and the perception of the neighbors. There was a remarkable coherence with a prior analysis [3] when the authors also analyzed the environmental sound scenario before and during the lockdown for each of the sensors. In the current work, we make a step forward and move to a much bigger city (i.e., Barcelona, Spain) and we correlate data from 70 acoustic sensors with the answers of the 119 volunteers who assessed soundscapes from different locations within the city.

In this study, the authors explore the possibility of deriving some human perceptions by means of objective data provided by a number of noise sensors in the city of Barcelona [4]. Objective data are compared with subjective data and possible correlations are pursued between sensors data and subjective responses gathered by a survey in the same city. All sensor data and subjective surveys were collected during the COVID-19 lockdown in 2020. Sensors are assembled by common main source of noise with the aim to check whether there is a clear correlation with the survey outcomes. Clusters of sensors are build and both the type and level of the noise are taken into account when comparing subjective soundscape assessment with objective acoustic data. Authors will also present an analysis on the acoustic satisfaction assessment prior and during the lockdown per type of main noise source, as well as an assessment of perceptual constructs for soundscapes for each cluster of contributions.

This paper is structured as follows. Section 2 shows the state-of-the-art of the sensor data collection, with special emphasis on the lockdown period. Section 3 details the lockdown restrictions in Barcelona, the data used in this work, the description of the studied areas and the data treatment performed. Next, Section 4 details several clusters of sensors in Barcelona with the comparison of the objective measurements and the subjective results. Finally, Section 5 offers a discussion on the results and on the strengths and limitations of the study. Section 6 details the conclusions of this work.

## 2. State-of-the-Art

The mobility and activity restrictions imposed by the COVID-19 pandemic provided, unintentionally, a unique scenario to evaluate how these restrictions affect the noise pollution levels and their perception by citizens. In the past three years, many studies have been conducted to assess the effects of the lockdown on different soundscapes. Most of the literature has focused on one of two approaches. On the one hand, some authors have chosen to analyze objective data collected by a sensor or a network of sensors during the lockdown in order to compare the results with normal pre-pandemic data. On the other hand, other researchers opted to conduct a survey about the perception of people during these same periods.

Most of the published articles on data gathered by sensors during the lockdown are from urban or sub-urban areas. However, several studies have also been conducted in other environments such as underwater soundscapes [5,6,7,8], marine soundscapes [9], offshore human activities [10] and airport surroundings [11]. Furthermore, most of the research conducted in populated areas focused mainly on determining the $L_{Aeq}$ reduction caused by the restrictions. Nonetheless, other changes in the soundscape have also been highlighted, e.g., in the San Francisco Bay Area, where a shift in the song frequency in some birds has been detected during the COVID-19 lockdown [12]. Apparently, the more favorable conditions caused by the drastic reduction in human activity and anthropogenic noise not only affected aspects like activity schedules, movement dynamics or exploratory behavior of different species [13] but also the vocalizations used in their songs in order to maximize communication distance in this new acoustic environment [12]. Another study [14] proved that the distribution of anomalous noise events and the intermittency ratio showed statistically significant differences in urban and suburban areas in Milan and Rome during the COVID-19 lockdown translating to a noticeable decrease in the negative impact of noise pollution in the population of both areas.

The geographical extent of these analyses varies from a single location in a city, e.g., Stockholm [15], to the combined contribution of seven of the major conurbations in India [16,17]. Lately, sensor networks consisting of sound meters have been deployed in many cities. Most of the recent publications that have studied changes in noise levels in 2020 have taken advantage of data collected from them. However, the scope of these networks differs significantly from one city to another ranging from 3 sound meters (as of the date of the study) in Montreal [18] to the impressive 70 sensors in Barcelona [4]. It is also worth noting that, in some cases, portable monitoring stations have been used [19,20] to gather noise data in a number of different locations when a permanent network of sensors was not available.

As expected, most of the literature verified a noteworthy reduction in the mean $L_{Aeq}$ level of noise pollution during the lockdown. Nonetheless, some notable exceptions have also been spotted. In a quiet residential area in the city of Kobe [21]. Noise levels were higher during the state of emergency declared, apropos of the COVID-19 disease. According to the author, this area experiences seasonal changes in noise levels, making it more difficult to correctly set target values of the acoustic environment planning by referring to the measured noise level during the shutdown. Also, in Boston [22], in one of the three protected areas assessed, located near a highway, sound levels were between 4 and 6 dB higher during the lockdown. The probable explanation provided by the authors is that in a scenario with reduced traffic, vehicles could travel faster, thus creating more noise. On the contrary, the two other protected areas, which were closer to the city center, experimented a decrease in 1–3 dB during the same period.

Several acoustic metrics have been chosen in the different studies, with $L_{Aeq}$, $L_{d}$ (stands for daytime equivalent noise level), $L_{n}$ (stands for night-time equivalent noise level) and $L_{den}$ (stands for day-night equivalent noise level) being the most widely used, which are consistent with some of the minimum indicators proposed by Asensio et al. [23]. Some authors offered the global average reduction of $L_{Aeq}$ during the lockdown in the city or region studied. The decrease in $L_{Aeq}$ is widespread but some differences are spotted according to the current restrictions present in each situation. Some of these changes in the mean $L_{Aeq}$ documented in the literature are 5.4 dBA in London [24] (ranging from 1.2 to 10.7 dBA), 5.1 dBA in the Ruhr Area (Germany) [25], 6–7 dBA in Montreal [18], 6–10 dBA in Monza (Italy) [26], a daily average peak drop of more than 4 dBA in Stockholm [15], 7 dBA in Rome and Milan [27] or 5.2–5.9 dBA during the peak of the restrictions in Barcelona [4].

This average reduction in the noise level is not necessarily consistent in all the locations where data were gathered. Some authors documented differences according to land use categories. In Rio [28], there was a noise reduction between 10 and 15 dBA in those areas with a predominance of human activities whereas there was no major reduction near major arteries. In Granada [29], the $L_{Aeq}$ variation ranged between 13.3 and 30.5 dBA depending on the location. Also, in seven Indian cities [16] the noise reduction ranged between 4 and 14 dBA for residential, industrial and commercial areas. Studies conducted in Madrid [30], the Ruhr Area [25] and Barcelona [4] among others also spotted differences according to the type of location.

Beyond the average $L_{Aeq}$ reduction, a comparison between day and night variations of the noise levels during the pandemic has also carried out done, e.g., in Buenos Aires [20], with decreases of 1.4–4.7 dBA during the day and 2.7–6.9 dBA during the night or in the Île-de-France region [31], with decreases in the road traffic noise of 4.6 dBA during the daytime and 7 dBA in the night. Differences in daily noise indicators have also been spotted in Barcelona [4], Girona [3], Dublin [32] or Madrid [30], among others, showing that time patterns were also affected during the lockdown. Moreover, other acoustic and even psychoacoustic metrics such as loudness and sharpness have also been calculated by some researchers, e.g., in London [24] where clustering of the 11 locations has been applied.

Regarding the people’s perception of the changes in the soundscape during the COVID-19 shutdown, there are two main approaches in the literature. On the one hand, some studies have surveyed people directly, asking about their perception of the soundscape with a guided set of questions. This is the case of a study set in Italy [33] where an 18-questions survey was answered by 323 participants and the results confirmed the expectation of a decrease in the noise pollution levels. Another similar work was conducted in France [31] where residents of the Île-de-France region perceived a significant reduction in the level of noise from a set of sources such as human activities, road traffic noise or airborne noise. In Argentina [34], a survey among 1371 social network users detected that people preferred the new acoustic environment caused by the COVID-19 lockdown. Also, in London [35,36] a mixed-method approach consisting of triangulating data from surveys and spontaneous descriptions offered by the participants (home workers) is being performed with the goal of finding associations between perception of the indoor soundscape and psychological well-being. Not only changes in residential soundscapes have been analyzed but also the impact on a historic soundscape such as the Berlin Wall Memorial [37] by means of soundwalks and informal interviews with staff members and tourists on the site. In Milan, ref. [38] the authors present a wide analysis of the changes in $L_{Aeq}$ in the city of Milan, evaluating the impact of the Anomalous Noise Events [39] in the different periods of the lockdown. Also, in [40] the situation was faced worldwide with questionnaires, including both indoors and outdoors, finding a clear improvement in the perception of the citizens facing the unpredictable situation of the pandemic. Another study conducted in Madrid on noise perception and related health effects during the lockdown presented a cross-sectional study by noise sources based on data collected from 582 participants who answered a questionnaire [41].

On the other hand, in some cases where people could not be surveyed, other approaches were selected. Mitchell et al. [42] developed a model to predict the soundscape pleasantness and eventfulness during the lockdown in London and Venice based on a database of previous binaural recordings and soundscape questionnaires and new recordings made during the pandemic. In the Basque Country [43], experts in soundscape and architecture listened to recordings taken between March and May 2020 and made two perceptual analyses, i.e., they annotated perceived sound events and assessed the pleasantness and eventfulness.

There are some precedents that combined an objective and a subjective approach in order to analyze the impact of the lockdown using a multidimensional approach such as in the city of Lorient, France [44], where data gathered from a network of sensors were used in addition to the citizen’s perception of the soundscape during 2019 and 2020, collected by two questionnaires, to improve the accuracy in describing changes in a sound environment. Finally, a systematic review on 119 studies about the perceptual change or the noise level change during the COVID-19 pandemic lockdown can be found in [45].

## 3. Methods and Data Gathered

The research presented in this work combines data of a diverse nature: (1) A-weighted equivalent sound pressure levels ($L_{Aeq}$) and other noise indices ($L_{d}$), ($L_{e}$) and ($L_{n}$) provided by a network of calibrated sound sensors deployed in Barcelona and (2) questionnaires answered by participants in the *Sons al Balcó* project including their perception of the soundscape around their dwellings both before and during the lockdown and details about the most annoying sounds spotted.

### 3.1. Lockdown Restrictions

In this subsection, the exact restrictions that were enforced in Barcelona during the lockdown and de-escalation stages are described to give context to the causes behind the decrease in noise levels and improvement of the acoustic comfort of citizens.

The lockdown period in Spain started on 14 March 2020, initially affecting only students and other professionals working in the education sector and ended on 3 May 2020. Within this period, there were weeks with a stricter confinement affecting all non-essential workers. However, the final days allowed children to go out for a walk and adults to practice sports outdoors in different time spots. In addition, shops, bars, restaurants, museums, libraries, sport facilities and leisure establishments were all closed. Public and private transport was highly reduced and only used to commute to the work place for the population still working.

After the lockdown began, the de-escalation process extended from 4 May 2020 to 17 June 2020. During these weeks, shops and other business started opening by appointment and with limited capacity. In the final stages of the de-escalation process, even the mobility to second residences was finally restored.

### 3.2. Questionnaire Design

The questionnaire was designed following some of the previous works of the team about perception and sound [46] related to health, and also other tests in the framework of former projects as LIFE-DYNAMAP [47], and analyzed and used to model the annoyance as in [48]. All the citizens answering the questionnaire were informed of the use that the research team would have from their answers and videos in terms of ethics and publication, and they signed a consent to use their information.

The main questions asked to citizens were related to the comparison of their soundscape before the lockdown and during the lockdown, as can be found in [49,50]. Some of the more relevant are the following:

- How do you describe the soundscape of your home, before the lockdown and during the lockdown?
- How do the following adjectives describe the soundscape you recorded? Loud, shrill, noisy, disturbing, sharp, exciting, calming, pleasant?
- Which sounds are present in the soundscape you recorded? Road traffic, plane, train, industry works, commercial activities, leisure activities, neighbors, pets, birds, water, vegetation?
- Please, indicate how much the former different sounds disturb you
- Compare the annoyance related to those sounds before and during the lockdown.

The researchers had the support of the X (formerly Twitter) accounts of both institutions involved in the *Sons al Balcó* project (ISGlobal and La Salle Campus Barcelona), and the dissemination of its own project X (formerly Twitter) account (@SonsalBalco). The researchers chose to map the soundscape of the lockdown in Catalonia due to the size, the potential population and the possibility of having contributions from both big cities like Barcelona, but also from small villages where the soundscape may not have changed so much during the lockdown. The citizens contributing were only asked to give a nickname, and did not require to register or log in to any platform, as all was conducted via web, which probably increased the participation but did not allow the team to contact the contributors after the data collection.

### 3.3. Data Collection Campaign

Several campaigns have been conducted to collect data in the *Sons al Balcó* project [49,51]. This present work is focused on data obtained from the first campaign performed in 2020 during the final stages of the lockdown caused by the COVID-19 pandemic and the initial stages of the de-escalation process. A socio-acoustic online questionnaire was designed to obtain perceptive data representative of the soundscapes across Catalonia. Some requirements were considered before launching the digital survey. First, LimeSurvey [52] was chosen as the web service platform to implement the online question-and-answer survey. The setting included different response formats and video uploading capacities. One of the main advantages of LimeSurvey compared to other survey applications is that it is an open-source solution that can be deployed to any server that supports it. Therefore, it is not constrained to the servers of the survey application provider. The specific implementation consisted on an Amazon EC2 cloud computing instance running a Bitnami Stack for LimeSurvey 4.2.3-0 on Ubuntu 16.04.6 LTS. Furthermore, for the purpose of reducing traffic, an Amazon S3 bucket was also applied to upload the recorded videos directly from the smartphones of the participants. Lastly, a Fine Uploader library running on EC2 was installed to manage and sign the requests allowing access to the aforementioned S3 bucket.

The questionnaire included different topics such as sociodemographic data, soundscape location and perceived quality, both before and during the confinement. Additionally, participants had to report the presence of different noise sources and their respective annoyance. These sound categories included different kinds of motorized traffic (automobiles, trains or planes), industry, construction works, commercial and recreational activities, neighborhood noise, pets, birds, water and vegetation. Further details on the survey can be found in [49].

### 3.4. Sensors Data

For this study, noise levels were obtained from the Wireless Acoustic Sensor Network (WASN) of Barcelona, which has already been used to conduct, in a previous work by the authors [4], a thorough analysis of several noise indices during the COVID-19 lockdown in the city.

**Figure 1:**
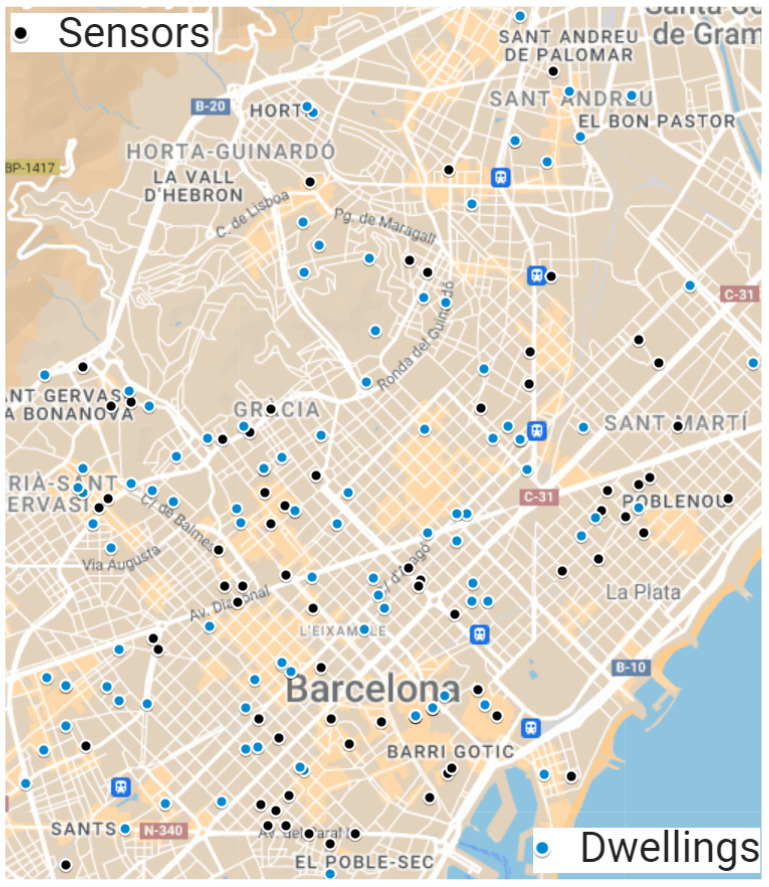
Location of the sensors and the assessed soundscapes during the 2020 *Sons al Balcó* campaign.

The WASN deployed in Barcelona (also named Barcelona Noise Monitoring Network) consists of 112 devices, 86 of which are sensors and 26 are sound level meters. These 86 sensors are placed for long-term analysis in several pre-analyzed places around the city. Since not all sensors worked properly during the lockdown, only 70 sensors out of the 86 deployed to conduct this study were analyzed. All the used sensors are CESVA’s TA120 Class 1 sound level meters. The location of these 70 sensors is depicted in Figure 1 along with the location of the assessed soundscapes. Furthermore, if we look at the distribution of noise sensors in Barcelona, there is a higher number of devices deployed in the city center and in leisure areas. This means that this network mainly monitors road traffic, commerce and leisure activities. A detailed depiction of the locations and maps can be found in [4]. Moreover, see [53,54] for more information about the Barcelona Noise Monitoring Network.

Sensors were active both in a normal pre-pandemic scenario and during the lockdown. Data from the first semester of 2018 and 2019 has been used as baseline levels for comparison with noise levels obtained during the lockdown. Most of the sensors were working 24 h a day during the studied periods and provided A-weighed equivalent sound pressure levels at one minute time resolution.

### 3.5. Description of the Studied Areas

As one of the main purposes of this study is to analyze the specific relationships between the decrease in noise levels and the improvement of the perceived acoustic comfort in different areas according to the main type of noise source, sensors have been manually grouped in different clusters. The first three groupings correspond to the majority of sensors deployed in areas where road traffic noise is the main source of noise exposure. They are divided into Heavy-Traffic Areas (sensors that measured mean values above 67.5 dBA during the baseline time-frame, i.e., the first semesters of 2018 and 2019), Moderate-Traffic Areas (mean values between 64.5 and 67.5 dBA) and Low-Traffic Areas (mean values below 64.5 dBA).

There are 16 sensors located in heavy-traffic areas. They are located in some of the main arteries of the city, which communicate different urban districts and connect with the entry and exit points to other cities in the metropolitan area. These streets are mainly used by people going and returning from their jobs and by delivery services and public transportation. Thus, they were less affected by the mobility restrictions, especially during the final stages of the lockdown. For this case, 22 respondents, both men and women, were considered to be in the scope of the selected sensors. The ages of the volunteers were between 19 and 72 years.

A total of 12 sensors are located in Moderate-Traffic Areas. These sensors are usually in mid-sized streets, often with multiple lanes. They are also occupied by people commuting using both private and public transportation. Even though road traffic is the predominant noise source present, these areas combine residential and office buildings with commerce and restaurants or even with some recreational facilities. For this cluster, 17 people were considered for the following results. They were both men and women from 32 to 69 years old.

There are sixteen sensors located in Low-Traffic Areas. They are located in quieter and smaller streets usually in the middle of residential buildings, with the occasional bar, grocery store or supermarket. The main sound source is traffic coming from the mobility of the neighbors. However, there is also commerce related noise and neighborhood noise. During weekends and especially Sundays, these locations are even quieter. In these sensor’ areas, there are 26 respondents whose age is between 28 and 71 years and they are both men and women.

The fourth grouping corresponds to sensors placed in SuperBlock areas which consist mostly in pacified streets. Barcelona’s SuperBlock project was conceived to reduce the road traffic in residential areas, mostly traffic derived from private vehicles [55]. The main project’s goal is to create greener and healthier urban public space. However, some of them are still under construction. In fact, the survey’s results reported that in 57.14% of the videos collected inside the area of influence of the SuperBlock sensors there were construction works spotted in the audios sent. A total of six sensors are located in SuperBlocks. In this case, seven participants evaluated soundscapes considered to be in the scope of SuperBlocks’ sensors. The age of the volunteers was between 23 and 50 years old and they were both men and women.

The next two clusters contain the sensors placed in areas were leisure activities, commercial activities and restaurants are the main source of noise exposure (both daytime and night-time leisure activities). Particularly, six sensors are located in areas where the main sound sources are related to Daytime Leisure activities and restaurants. Most of these sensors are located in small squares in the middle of residential areas in different city districts. Road traffic is limited or nonexistent. The main source of noise comes from the passersby, pedestrians, tourists, playgrounds and restaurants. Occasionally, there is also some commercial and nightlife activity in these areas. For this cluster of sensors, 24 people answered the questionnaire.

Next, there are nine sensors located in areas where the main noise source comes from Night-Time Leisure activities. They are in neighborhoods full of bars, pubs and restaurants and it is especially active on afternoons, evenings and nights all along the week with lots of interaction from tourists and students. Trading activity also produces a moderate activity during the day. In this area, there were 6 volunteers that fit the profile, who were both men and women from 32 to 50 years old.

From the 70 sensors studied, 65 are distributed among these 6 types of locations. The other 5 sensors are placed in industrial areas or parks and 5 videos were collected inside the area of influence of those 5 sensors. However, they are too few to extract any meaningful aggregated information from them and they have not been included in the clustered study.

### 3.6. Data Treatment

While data from different sensors were gathered during the 2020 lockdown period, a socio-acoustic digital participatory survey was also performed. It aimed to gather the positive and negative perception of noise experienced from home before and during the lockdown. In this survey, the respondents were told to record a video of their sound environment (representative of a typical daily soundscape during the lockdown) and answer a questionnaire about their perception. In total, 366 volunteers from 132 different locations completed the questionnaire and uploaded their videos. One of these contributions had to be discarded because the location was ambiguous. Almost 40% out of the 365 accepted participants came from cities with a WASN deployed. Twenty-two were from Girona and preliminary results on their analysis have already been published by authors [2]. This present work will be anchored in the largest portion of volunteers (119) that described soundscapes from Barcelona in the survey. The contributors’ profile was both men and women between 29 and 86 years old.

To ensure the reliability of the responses, the videos recorded were manually labeled and the sound events reported by volunteers were compared with the sound events spotted by annotators. In addition, only the questionnaires with the essential data correctly provided were accepted for this study, i.e., the exact location of the soundscape and the general assessment of the soundscape both during and before the lockdown.

Authors opted for a clustered approach to perform the study. As stated in Section 3.5, both sensors and assessed soundscapes are being manually grouped in clusters according to the predominant noise source and density of traffic. This approach aims to offer more insight in which kind of noise sources regulation have a greater impact in the acoustic satisfaction of citizenship.

Contributions from Barcelona were assigned to the area of influence of their nearest sensor. Several preliminary experiments were performed with accepted radii from 400 m to 1 km with comparable results. Finally, contributions from a maximum of 1 km distant to the sensor were taken into account for the analysis, assuming that farther contributions may have a very different soundscape and even different urban sound environment (leisure, schools, traffic, etc.). Schafer [56] already explains that focus listening with its implication of distance separating the listener from the sound event is disintegrating before the sound walls, so lo-fi soundscapes do not have perspective, but mask the listener with a constant presence. Distances between the sensors and the location of the assessed soundscapes were evaluated using the Haversine formula [57], an easy to implement method for obtaining optimal approximations of distances over a circle when the longitude and latitude are known.

Each one of the studied sensors provided $L_{Aeq}$ noise levels at a one-minute time resolution. The mean values for the different noise indices ($L_{Aeq}$, $L_{d}$, $L_{e}$ and $L_{n}$) are computed for each sensor both for the lockdown period (from 14 March 2020 to 3 May 2020) and for the baseline time-frame (first semesters of 2018 and 2019). After that, the mean noise indices corresponding to sensors from the same cluster are also averaged to obtain the final mean noise indices for the given cluster. Variances for the measured $L_{Aeq}$ decreases will also be calculated.

Volunteers assessed the acoustic comfort around their dwellings both for the lockdown period and for the pre-lockdown period using a Likert Scale [58] (Very Negative, Negative, Neutral, Positive and Very Positive). This Likert Scale is converted to a 5-point scale (1 to 5). After that, both ratings are compared to obtain the soundscape rating improvement for each dwelling. The average and the variance for all the soundscape rating improvements of the dwellings included in the same cluster are subsequently computed.

Subsequently, the $L_{Aeq}$ decrease for each one of the studied clusters will be compared with the reported improvement of the subjective acoustic comfort (soundscape rating by volunteers). The hypothesis is that a general alignment will be found between both objective and subjective data but that there will be significant differences between some of the clusters, especially related to the different predominant noise sources, e.g., traffic or leisure.

The previous statistical results will be complemented with a more nuanced study, both quantitative and qualitative, of the daily noise indices and other answers provided in the survey (for each cluster). Specifically, distinct intra-day pattern variations in the mean sound pressure level for each cluster will be described. Also, the main types of actual noise sources spotted in the area will be commented. The specific subjective assessment both before and during the lockdown (using a Likert scale) for the overall quality of the soundscape and the assessment of several perceptual constructs will also help give more insight into the appraisal of the soundscapes and the possible outlier opinions included.

## 4. Results

This section offers a detailed description of the comparison between the objective acoustic data gathered by the sensors in Barcelona and the subjective data collected from the citizen science campaign. An individual analysis and interpretation has been conducted for each one of the six clusters described in Section 3.5.

### 4.1. Barcelona General Results

In this section, we describe the most relevant sensors results in Barcelona together with the subjective contributions of the citizens. The goal is to show whether the conclusions reached on previous research studies, ref. [4] and summarized below might show some qualitative coincidence with the answers to the poll.

Barcelona experimented a significant drop in noise level during the lockdown stages in 2020. The decrease was especially steep during the stages with stricter mobility and activity constraints and during the night hours. This decline has to be put in context as Barcelona was already showing a mild but steady noise reduction trend in most of its sensors from 2018 to the first months of 2020, probably due to the pacifying efforts being implemented in recent years by the local administration.

Although all areas of the city produced lower noise levels during the lockdown stages, they were not equally affected. Areas with heavy traffic experienced lower noise reduction than areas with moderate traffic, mainly during the day. In residential and low-traffic areas, the reduction was more restrained because the pre-lockdown noise levels were also lower. As for the other sources of studied noise, they also showed differences. On the one hand, daytime leisure, restaurant areas and nightlife areas were among the most affected, with distinct intraday noise variation. Nightlife areas took a huge plunge during the evening and night-time frames, whereas daytime leisure and restaurant areas were more affected during afternoons and evenings. Furthermore, Superblocks and shopping areas presented a similar drop irrespective of the hour of the day. On the other hand, industrial and services areas were among the less affected by the restrictions, basically during the morning hours.

Table 1 compares the mean improvement in the $L_{Aeq}$ level during the lockdown with the subjective improvement perceived by participants in the *Sons al Balcó* project during the same time-frame. Data collected came from 70 sensors deployed in Barcelona during the lockdown and from 119 surveys answered by Barcelona citizens. Each row in the table corresponds to the different clusters described in Section 3.5. In the second column, the total number of sensors included in each investigated area is shown.

The third column in Table 1 reports the number of videos and questionnaires collected inside the area of influence of the corresponding sensors. The videos have been assigned to the area of influence of a single sensor, the nearest one. Only videos collected within a 1 km radius from the nearest sensor have been considered. The mean distance in km from the locations where the videos were recorded to the nearest sensors are shown in Column 4. Column 5 shows the mean dip in the $L_{Aeq}$ level during the lockdown in 2020 compared to the same levels measured during the same weeks in 2018 and 2019. Column 6 contains the variance of the $L_{Aeq}$ decrease for the different sensors. Column 7 shows the perceived mean improvement in the soundscape rating (and the percentage of improvement over the original rating), after converting the original Likert scale used by participants to numerical values from 1 to 5. Finally, Column 8 includes the variance of the soundscape rating improvement according to the volunteers.

Even though the mean $L_{Aeq}$ improvement is higher for the SuperBlocks and Leisure areas than for the areas mostly affected by road traffic noise, the perceived subjective improvement is higher for those areas where road traffic noise is predominant. That hints at the fact that road traffic noise is especially annoying for most of the participants. Reducing road traffic noise exposure has a greater impact on the general subjective assessment of the improvement than reducing other types of noises.

Focusing on the three different traffic areas (heavy, moderate and low), there is a correlation between the objective mean improvement in the $L_{Aeq}$ levels and the subjective mean improvement reported by contributors. Moderate-Traffic Areas are where the improvement is higher, followed by Heavy-Traffic Areas and, finally, Low-Traffic Areas.

SuperBlocks experienced a high drop in the $L_{Aeq}$ level that was translated to a more modest improvement in the subjective assessment. One of the reasons is that construction works were already resumed by the time the videos were collected and they were especially abundant inside the areas of influence of the SuperBlock sensors.

Leisure areas were especially affected by the activity restrictions conducting to mean $L_{Aeq}$ drops of more than 7.3 dB during the lockdown. However, the subjective assessment only improved by 33 to 35% (which pales in comparison to the improvement in road-traffic areas). The main reason for that is that the original (before the lockdown) subjective assessment of the soundscapes collected in those areas was higher than the subjective assessment of the road-traffic-exposed areas. Leisure areas had an original mean rating of 3.2 points. Therefore, it was virtually impossible to achieve the improvements reported in the road-traffic-exposed areas.

The improvement for both the objective measurement and the subjective perception was slightly higher for the nightlife areas than for the Day-time Leisure Areas showing, again, a correlation between the subjective perception and the objective reduction in sound levels.

Aggregated data in the last row of Table 1 include all the sensors active in Barcelona during the lockdown and all the valid contributions received during the 2020 campaign of *Sons al Balcó* from the city of Barcelona, even the ones beyond the 1 km threshold.

**Figure 2:**
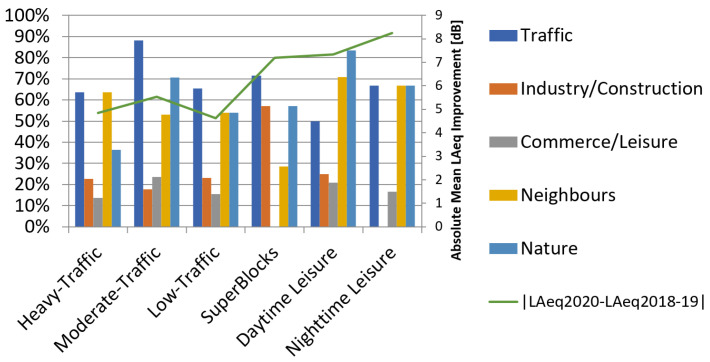
Percentage of appearance of sound events reported by type of area compared with the $L_{Aeq}$ improvement during the lockdown.

Figure 2 shows the presence of several kinds of noise sources and other sound events in the received videos sorted by the type of area in which the nearest sensor is circumscribed. The percentage depicted in the figure is related to the number of contributions where participants spotted each of the sound events compared with the total of contributions for each cluster. The absolute mean $L_{Aeq}$ improvement during the lockdown compared to the mean noise level for the two previous years is also represented in the figure for comparison purposes. Noise sources have been grouped in five categories: (a) Traffic, which includes all types of motorized traffic; (b) Industry/Construction, which includes noises from both industrial sources and construction sites; (c) Commerce/Leisure, which includes noises from recreational activities, restaurants and shopping areas; (d) Neighbors, which includes neighborhood noise and pets and (e) Nature, which includes sound events related to nature elements and wildlife (mainly water, vegetation and birds).

The most prevalent sound in most of the clusters is traffic noise, which appears in 50% or more of the videos independently of the type of area where the nearest sensor is located. Road traffic noise prevalence is higher in those areas where the $L_{Aeq}$ improvement caused by the restrictions of the lockdown was less significant. That hints at the fact that road traffic noise is one of the main contributors to the global $L_{Aeq}$ in urban locations. On the contrary, nature sounds appear more frequently in areas where the $L_{Aeq}$ took a steeper dip. That is caused because some sound sources such as birds that are easily squelched in noisy soundscapes became apparent when the louder noise sources decreased.

It is also noteworthy that in the lockdown context, the third sound event most prevalent after traffic noise and nature related sounds is neighborhood noise, which was spotted in above 50% of the videos for all the studied groups except for SuperBlocks. In contrast, industry and construction noise and, especially, leisure and commerce noise was greatly decreased. These three categories (neighbors, industry/construction and commerce/leisure) appear to be independent of the measured $L_{Aeq}$ improvement.

A more detailed analysis of each type of urban area is conducted in the subsequent subsections.

#### 4.1.1. Sensors in Heavy-Traffic Areas

As seen in Table 2, mean pre-lockdown daily noise indices for theses sensors were significantly higher than those from sensors of other clusters, with more than 70 dB during the day and almost 65 dB during the night. Even though indices were clearly improved during the lockdown, they remained notably high when compared to sensors in other areas. Furthermore, the improvement caused by the restrictions in this group of sensors was inferior to all the other studied groupings during days and evenings and the second-to-smallest during nights.

The soundscape evaluation before the lockdown was generally poor, with 50% of participants rating it as “Negative” or “Very Negative” (Table 3). In general, contributions located in areas where road traffic is the main noise source reported more deteriorated soundscapes (before the lockdown) than areas where the predominant noise source is different (always according to the opinions reported in the surveys). In contrast, during the lockdown the subjective evaluation of the quality of the soundscape experienced one of the higher boosts compared to other clusters. As observed in Table 3, all the 50% negative assessments pre-lockdown changed to neutral or positive assessments. In addition, 63.64% of dwellings considered to have “Very Positive” soundscapes during the lockdown is the second-to-highest figure for the studied clusters only after SuperBlocks.

Comparing the huge improvement in the subjective acoustic satisfaction assessment (Table 3) with the rather modest reduction in the levels in noise indices (Table 2), it seems that the original pre-lockdown noise levels being as high was a critical cause of dissatisfaction and that the modest reduction they experienced during the lockdown was enough to significantly change the perception of the soundscape.

**Figure 3:**
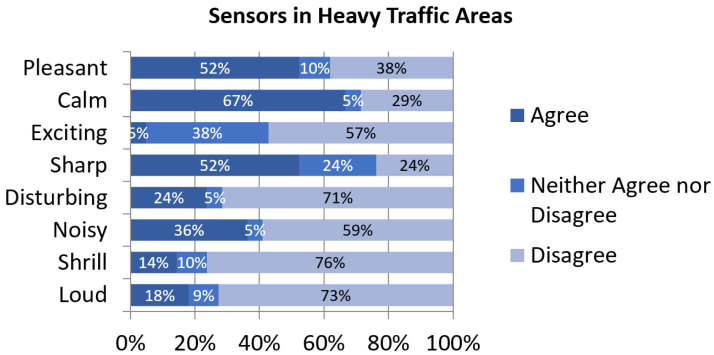
Assessment of perceptual constructs for soundscapes near sensors in Heavy-Traffic Areas.

The perceptual constructs’ assessment is quite heterogeneous. However, there is general consensus in all the studied clusters that positive perceptual constructs (with the exception of excitement) are more representative of the studied soundscapes than negative perceptual constructs. That being said, 36% of respondents in this cluster find their soundscape noisy and 23% find it disturbing (Figure 3). These percentages are generally higher than in other clusters (with the exception of Low Traffic) which is consistent with the higher noise indices in Heavy-Traffic Areas. The main noise sources reported in this group are road traffic and neighborhood noise and the most annoying, according to survey results, is road traffic, which is coherent with sensors located in Heavy-Traffic Areas.

#### 4.1.2. Sensors in Moderate-Traffic Areas

Before the lockdown, the mean noise indices in this cluster (Table 4) were approximately 3 dB lower than in the Heavy Traffic cluster (Table 2) but significantly higher than in Low-Traffic Areas (see Section 4.1.3). However, from the three traffic focused clusters, they are the ones that showed a larger decrease during the lockdown.

According to the answers, there was a huge improvement in the sound environment of these sensors when comparing before and after the lockdown periods. In fact, it is the cluster that showed a bigger amelioration of the global subjective rating of the soundscape during the lockdown (Table 1). Before the lockdown, more than 50% of the respondents rated their dwelling’s soundscape as “Negative” or “Very Negative” and not a single participant considered it to be “Very Positive” (Table 5). However, during the lockdown, almost 95% of them considered that the soundscape was “Positive” or “Very Positive” with no reported cases of negative assessments. Objective and subjective data are clearly aligned for the traffic clusters. Moderate-Traffic Areas show both the steeper dip in the noise levels during the lockdown (compared to Low and Heavy-Traffic Zones) and also show the bigger improvement in the acoustic satisfaction degree of its inhabitants.

This vastly positive evaluation of the soundscapes could be explained by the fact that road traffic was drastically reduced and although it progressively increased in the de-escalation process, it never recovered the former density, as mentioned in [4]. In fact, even though road traffic is the most spotted sound event in Moderate-Traffic Areas, appearing in almost 90% of the recordings (Figure 2), the reported annoyance caused by road traffic noise is lower than the annoyance caused by the less prevalent leisure and commerce activities and construction works in the area. Again, the fact that the most predominant sound source is not considered especially annoying in the surveys is consistent with the reduced noise indices during the lockdown.

**Figure 4:**
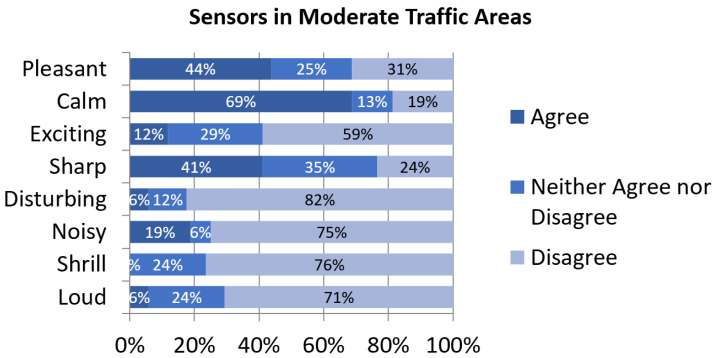
Assessment of perceptual constructs for soundscapes near sensors in Moderate-Traffic Areas.

In general terms, perceptual constructs’ assessment in this cluster is similar to the other groups. Participants agree more regarding the representation of positive constructs such as calmness or pleasantness than in negative constructs (Figure 4). It is to be noted, though, that the exceeding percentage of dissent for disturbance, noisiness and shrillness is considerably higher than in the other traffic-related clusters.

#### 4.1.3. Sensors in Low-Traffic Areas

Noise indices for this group of sensors were the lowest during 2018 and 2019 (Table 6). However, they experienced a milder decline during the lockdown and, in fact, they were no longer the lowest during the restrictions, surpassed by Day-time Leisure Areas.

The initial pre-lockdown assessment of the soundscapes in the surveys amounts to fewer negative evaluations overall, with around 42% of “Negative” or “Very Negative” appraisals compared to the 50% or more of the other clusters related to traffic noise (Table 7). They also achieve a substantial improvement during the lockdown. However, it is the only cluster where some soundscapes rated “Very Negative” remain. Results seem a little inconsistent with other clusters but a more detailed analysis detected some outlier opinions among the respondents which is consistent with the higher than normal variance in the soundscape rating improvement reported in Table 1. Surprisingly, about 10% of the respondents considered that the quality of the soundscape had deteriorated during the lockdown. Analyzing the responses, construction works were reported near the locations of two of the dissenting citizens, which is the most probable cause for this exceptional dissatisfaction with the acoustic environment. In fact, in a similar way to Moderate-Traffic Areas, traffic was the most reported noise source among contributors but it was also significantly less annoying than construction and commerce activities (much less prevalent in the studied soundscapes).

**Figure 5:**
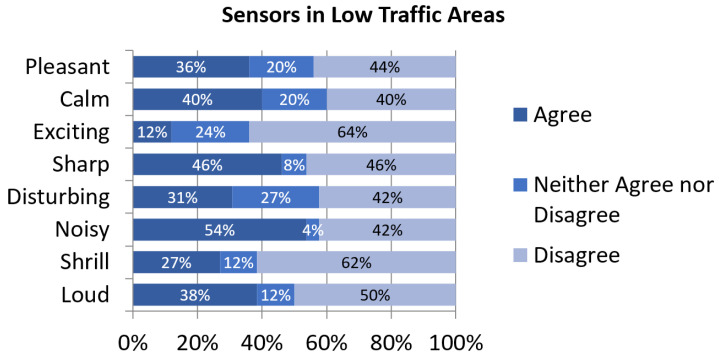
Assessment of perceptual constructs for soundscapes near sensors in Low-Traffic Areas.

Perceptual constructs’ assessment in Low-Traffic Areas shows a wide range of dissenting opinions among citizens (Figure 5). It is the only cluster where positive and negative perceptual constructs are similarly rated. Surprisingly, in these quieter parts of the city where noise indices are lower, *noisy* and *loud* are more often depicted as adequate adjectives than *pleasant*. Again, an explanation can be found in many construction works resuming their activity in the final stages of the lockdown after a long period of virtually no noise in the surroundings. Furthermore, inhabitants of these quieter areas are less used to high levels of noise pollution and their expectations may be more demanding.

#### 4.1.4. Sensors in SuperBlock Areas

One would expect that sensors in this cluster would provide similar noise indices to those of the Low-Traffic Areas as they are both mainly located in residential quieter zones. However, noise levels in Table 8 are significantly higher, similar to the indices of Moderate-Traffic Areas. There are two causes that explain these figures. First, some of the SuperBlock sensors are really located near the bordering streets of the pacified area where there is an exceeding traffic density caused by the mobility restrictions inside the SuperBlock and do not really represent the noise levels present in the inner buildings. Second, and even more relevant, intensive construction works were being executed in SuperBlocks (see Figure 2) in order to convert spaces previously dedicated to motor traffic to pedestrian areas. As a consequence, there are significant differences between the reported decrease in the noise indices during the lockdown among the sensors included in SuperBlocks as it can be observed by the overly high value of the variance reported in Table 1 which is accentuated by the reduced number of sensors available in the cluster.

The analysis of the subjective results for this cluster has to be taken as a qualitative approximation with limited reliability due to the few contributors who reported soundscapes in SuperBlocks areas and the higher variance also detected in the subjective aprraisals. First, it should be noted that a single participant rated the soundscape around his dwelling as “Negative” during the lockdown (Table 9). In fact, this citizen considered that the quality of the soundscape had worsened compared to the pre-lockdown scenario. Again, construction works are the probable cause for his outlier opinion according to his answers to the survey. The other six participants agree that the soundscape quality vastly improved in 2020 giving, in fact, the highest percentage of “Very Positive” ratings among all the studied clusters. This overly elevated degree of acoustic satisfaction is aligned with the $L_{d}$ variation during the lockdown which, at −7.22 dB, is significantly higher than in the other clusters.

**Figure 6:**
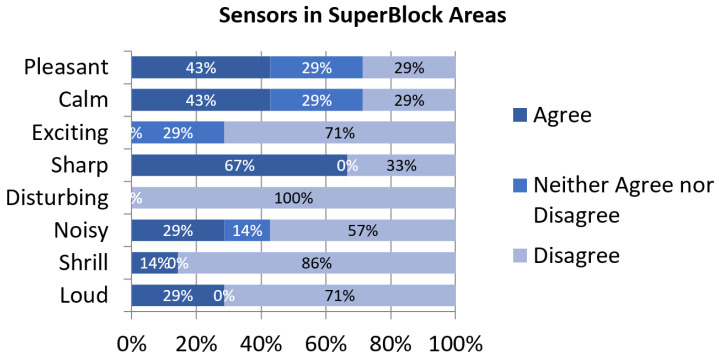
Assessment of perceptual constructs for soundscapes near sensors in SuperBlocks areas.

The evaluation of perceptual constructs by inhabitants of SuperBlocks’ surroundings is similar to the other clusters. However, it is noteworthy that all of the volunteers disagreed with their soundscape being *disturbing* (Figure 6). It may be related to the total absence of commerce or leisure-related sounds reported in this subset of questionnaires, as shown in Figure 2.

#### 4.1.5. Sensors in Day-Time Leisure Areas

Intraday patterns for this group of sensors are significantly different from road-traffic-dominated domains. Noise levels increase during the afternoon after class and after finishing the working day and remain high through the evening, taking into account that in Spain dinner time can easily extend to 23 h. Therefore, in a normal scenario, $L_{e}$ is especially elevated, usually surpassing $L_{d}$, as seen in Table 10. As leisure activities and restaurants were severely affected by the restrictions, this cluster presents the lower noise indices during the lockdown, including the steeper drop in $L_{e}$. Owing to that, the noise indices pattern during the confinement followed the usual trend in other areas where $L_{d}$ towers over both $L_{e}$ and $L_{n}$.

The acoustic satisfaction of the respondents for the pre-pandemic soundscape was fair-to-middling with a majority of people considering it neither positive nor negative (Table 11). On the contrary, a vast percentage of people changed their appraisal to “Positive” or “Very Positive” when the restrictions remained in force. This improvement completely aligned with the drastic decrease in the $L_{e}$ index, which is the most representative of the noise caused by daytime leisure activities and restaurants as it has been already stated.

**Figure 7:**
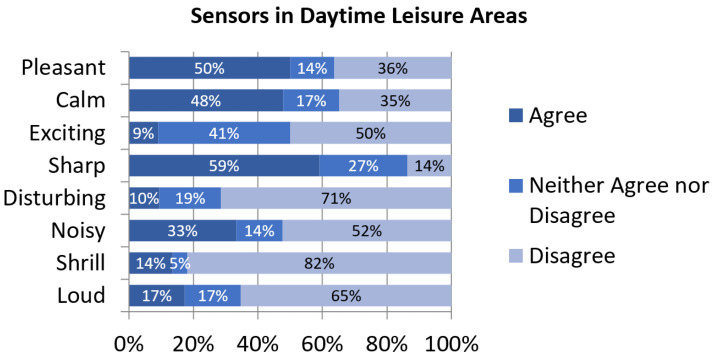
Assessment of perceptual constructs for soundscapes near sensors in Day-time Leisure Areas.

Sound events spotted in Day-time Leisure Areas when the campaign was performed are significantly different from other areas. Specifically, it is the only grouping where nature-originated sounds were reported in more than 80% of the contributions, surpassing road traffic noise. In fact, it is the only cluster where nature sound categories are more prevalent than any of the other sound classes, followed by neighborhood noise (Figure 2). Again, this is consistent with the fact that noise indices were lower than in other areas in the period affected by the restrictions. These exceedingly low indices also contribute to a sharper soundscape, which is the perceptual construct more people agree on in the survey (Figure 7). As for the other perceptual constructs, their assessment is along the lines of most of the other clusters.

#### 4.1.6. Sensors in Night-Time Leisure Areas

Noise indices in Night-Time Leisure Areas also follow intraday patterns significantly different from road traffic areas with elevated $L_{e}$ and $L_{n}$ which are consistent with the peak hours of night-time activity. In fact, $L_{n}$ in this cluster is especially high, only second to the levels of Heavy Traffic sensors (Table 12). These areas were especially affected during the lockdown due to the curfew and mobility restrictions, which cancelled most of the activity. Therefore, it is not surprising that the $L_{n}$ collapsed from 62.74 dB to 49.49 dB, the most impressive drop among all indices. Also noticeable is the decrease for the $L_{e}$, only surpassed by the drop in the Daytime Leisure cluster.

As in the case for SuperBlocks, the number of contributions is limited and results of this cluster should be complemented with additional data when it is available. It is the only cluster of contributions where there was a 100% consensus regarding the fact that all the soundscapes where deemed “Positive” or “Very Positive” during the lockdown (Table 13). It is also noteworthy that there were not negative assessments of the soundscapes in the pre-lockdown scenario; the majority of participants considered their acoustic environment neither positive nor negative, which is aligned with the average noise indices detected in the 2018–19 period.

**Figure 8:**
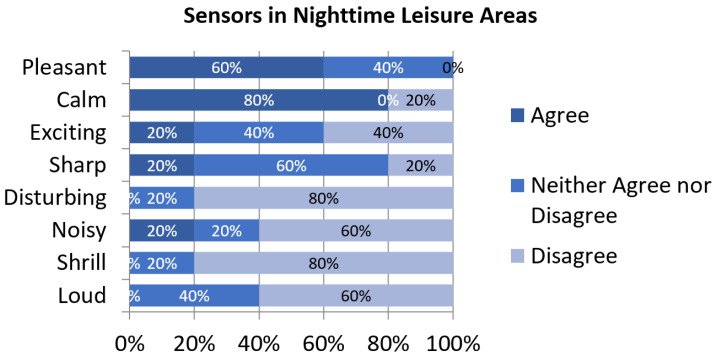
Assessment of perceptual constructs for soundscapes near sensors in Night-Time Leisure Areas.

Typical noise sources almost disappeared during the lockdown, with very few instances of leisure or commerce-related noise reported (Figure 2). In addition, there were not any industry or construction work noises in the surroundings of the participants. The predominant sound events reported were traffic noise, neighborhood noise and nature sounds with the same prevalence. These changes in the soundscape elements combined with very low noise levels are correlated with a higher consensus on some of the positive perceptual constructs (Figure 8). Inhabitants of these areas are the ones that mostly agree with their surroundings being *calm* and they also majorly agree with it being *pleasant*. Also, in most negative perceptual constructs there is not a single respondent that agrees with them as valid describers of their acoustic environment.

## 5. Discussion

Results in Table 1 showed a strong alignment between the mean $L_{Aeq}$ improvement during the lockdown for each cluster and the correspondent mean soundscape rating improvement for the same period. In fact, correlation between Columns 5 and 6 of Table 1 are 97.43% for the first three clusters dedicated to road-traffic noise and 58.32% for the next three clusters with different predominant noise sources. Therefore, the clustered approach seems to be appropriate for the purpose of comparing objective and subjective data.

On the contrary, comparing individual improvements for each one of the videos’ subjective assessment with the individual drops in the $L_{Aeq}$ observed in the nearest sensor provided a very low correlation of barely 4%. This very modest correlation rises significantly when the predominant noise source in the area is taken into account. If this individual comparison is performed only in areas where road-traffic is predominant (which include 65 of the contributors) the correlation is more than six times higher (26.44%). Also, the individual comparison performed only in areas where leisure noise is predominant (30 participants in total) gave an even higher correlation of 31.72%.

These figures show that with the clustered point of view the relationship between both sensor data and perception is clearly stronger than when the individual soundscapes are evaluated. In addition, they also highlight the importance of taking into account the predominant noise source present in the surroundings of a given soundscape along with the noise levels to maximize the alignment with its perceived quality.

The clustered approach offers a global perspective of the relationship between the perception of the soundscapes during the lockdown and sensor data. However, there are some dissenting opinions that do not align with the general appraisal of the soundscapes during the lockdown. Three of the participants rated their degree of acoustic satisfaction during the confinement lower than in the pre-lockdown period and another one kept the “Very Negative” assessment both before and during the lockdown. Three of these dissenting opinions are explained by the presence of construction works which resumed exactly when the survey took place. The other outlier assessment seems less coherent after analyzing all the answers provided in the survey by this specific contributor. Even though there are only four dissenting opinions, they have a significant impact in the correlation between the decreasing of the $L_{Aeq}$ level and the improvement of the subjective perception due to the limited total number of contributions available.

In areas where road-traffic noise is predominant, daily noise indices follow a clearly defined pattern where $L_{d}$ is higher than $L_{e}$ and $L_{e}$ is higher than $L_{n}$. That does not happen in areas where the primary noise sources are leisure activities. In Day-time Leisure Areas, the higher level usually corresponds to $L_{e}$, followed by $L_{d}$ and $L_{n}$. In Night-time Leisure Areas, the pattern is similar but night levels are significantly higher and can be very close to $L_{d}$ and $L_{e}$ levels. Therefore, lockdown regulations affected differently the noise indices depending on the type of area. For areas dominated by road traffic, daily noise indices were similarly decreased and correlated with the $L_{Aeq}$ drop. For that reason, an analysis based only on $L_{Aeq}$ may be sufficient. However, for areas dominated by leisure noise, $L_{e}$ and $L_{n}$ experienced especially higher drops compared to $L_{d}$. In consequence; it is convenient to take into account daily noise indices to obtain a clearer picture of the type of noise sources more affected and the correspondent changes in citizens’ appraisal of their acoustic environment.

There are some strengths in this study that are worth mentioning. Available objective data are especially sound because the Barcelona Noise Monitoring Network is an exceptionally large WASN with up to 70 working sound sensors during the lockdown. This vast amount of sensors facilitated that most assessed soundscapes were located relatively near a sensor (normally within 300 to 500 m depending on the cluster).

Most of the surveys in this project were answered in May 2020 during the initial stages of the de-escalation process (the last one accepted was from 12 June). Therefore, the time elapsed from the stricter lockdown in April was relatively short and there were still activity and mobility restrictions in force. In contrast, other studies in the same field found in the literature collected data in the later stages of the de-escalation process, which can have a significant effect on the subjective appraisal of the acoustic environment.

There are also limitations in this work that must be stated. Available subjective data are the main constraint of this work. It is true that more than one hundred of surveys were included in the comparison (which is a number consistent with other similar questionnaire-based studies published [59,60,61]). However, for attaining higher representation, a larger amount of opinions should be taken into account.

Not all clusters include the same number of sensors. The clusters with fewer sensors usually have a higher variance in the reported decrease in noise levels that have to be considered. In fact, results for the SuperBlocks cluster are not significant because they are based both in a very limited number of sensors and in a reduced set of assessed soundscapes. In addition, the reported noise and soundscape rating improvements have a very high variance affecting the reliability of the comparison.

There are no significant biases in gender and age between the respondents. However, there is a clear bias in their educational level as 87.72% of the participants had a university degree, which is an usual bias in this type of research projects [41]. Also, the study is focused in Barcelona, which is a big metropolis. Results may not be representative of smaller and less populated urban areas.

## 6. Conclusions

In this research, authors have compared objective data from acoustic sensors deployed in several districts in a big city (i.e., Barcelona, Spain) with the answers obtained from a questionnaire assessing the soundscapes in the surroundings of the sensors as a continuation of the preliminary research conducted in Girona, Spain, by the same researchers [2]. The comparison clearly manifests a coincidence between the reported soundscape quality during the lockdown and the improved level of noise indices collected by the sensors.

In a number of locations, especially in areas with higher levels of road-traffic noise, the reduction in the overall noise level highlighted other noise sources not perceived before the lockdown, such as Birds and Neighbors. This phenomenon is proved by both the gathered data from the sensors and the answers given by the participants. However, the new noise sources were not perceived as a nuisance but as pleasant. Therefore, according to the respondents, this change in the sound environment after confinement was for the better. That implies that a decrease in the road traffic noise has two direct benefits. Not only does it provides lower noise indices but it also changes the soundscape constitution from a scenario where only annoying noise sources are spotted (usually related to motorized traffic) to a scenario where both annoying and pleasant sound events are equally represented (both traffic and nature related).

The clustering of the contributions according to different kinds of predominant noise sources highlighted significant differences among them. Road traffic areas experienced the highest increases in the subjective evaluation by the volunteers even though the corresponding decrease in the noise indices was smaller in the same zones. On the contrary, in the surroundings of spots with predominance of leisure noise, a major drop in the noise indices translated into a milder increase in the subjective appraisal of the soundscape. This hints at the fact that road traffic noise is especially annoying. Therefore, its reduction has a greater effect on the acoustic comfort of citizens than other sound sources. These results have a direct impact both in environmental governance and urban planning. Measures taken by the public administration in order to increase the acoustic comfort of the residents should consider giving priority to the decrease in road traffic noise among other noise sources as they will be more effective. Accordingly, keeping road traffic noise as isolated as possible from dwellings and working places should also be a priority for urban planners.

Also, construction work has an important impact both on the noise levels and in the negative appraisal of the soundscape by those living nearby. On the contrary, other noise sources such as neighborhood noise or commercial noise are not as clearly correlated. For that reason, it is highly advisable to take into account the type of noise sources in addition to the measured noise levels when modeling the soundscape perception for prediction purposes.

The assessment of perceptual constructs by participants is heterogeneous, especially in comparison with the global assessment of the quality of the soundscape. They are generally aligned with the overall improvement reported with the objective data provided with sensors. In fact, there is a general consensus in the predominance of positive perceptual constructs over negative ones. However, differences among the various positive perceptual constructs are less correlated with the noise indices. Likewise, individual percentages for each one of the negative perceptual constructs are not exactly correlated with variations of the objective noise levels. Therefore, to obtain information more aligned with the objective data, a combined analysis of the perceptual constructs is more recommended than an individual approach.

In the future, the authors plan to start the automatic detection of sounds and objects in the videos uploaded by citizens in the framework of the still opened project *Sons al Balcó*. After the lockdown, the project has been conducted into local collecting campaigns, as the ones conducted in Sabadell (https://sonsalbalco.salle.url.edu/sonsdesabadell/, last accessed 24 August 2022) and in Granollers (https://sonsalbalco.salle.url.edu/sonsdegranollers/, last accessed 24 August 2022). The video and questionnaires collection has been wider in smaller cities, and this fact opens the possibility of starting the work in designing the indicators to evaluate both the objective and calibrated measurements ($L_{Aeq}$, etc.) with the annoyance and pleasantness evaluated subjectively via questionnaires. The automatic detection of objects and sounds in the videos would increase substantially the information for each citizen’s contribution and enrich the indicators design. The next steps are focused on using more data coming from citizen participation to start the definition of indicators useful for administration and researchers, combining objective and subjective evaluation of noise and soundscapes, and finally, attempting to predict the subjective perception of a soundscape using several indicators including WASN-based $L_{Aeq}$ levels.

## Figures and Tables

**Table 1:** Comparative of the mean improvement in the $L_{Aeq}$ level during the lockdown and the mean subjective improvement in the soundscape rating (acoustic satisfaction) reported by the participants in the *Sons al Balcó* project during the same time-frame.

Type of Location	Num. of Sensors	Num. of Soundscapes	Mean Distance Sensor-Video [km]	Mean $L_{\mathrm{Aeq}}$ Decrease	Variance $L_{\mathrm{Aeq}}$ Decrease	Mean Soundscape Rating Improvement	Variance Soundscape Rating Improvement
Heavy Traffic	16	22	0.39	−4.84	2.14	+1.77 (62.77%)	1.42
Moderate Traffic	12	17	0.31	−5.54	1.21	+2.12 (92.58%)	1.24
Low Traffic	16	26	0.47	−4.63	1.07	+1.5 (58.14%)	3.06
SuperBlocks	6	7	0.4	−7.2	15.98	+1.14 (34.65%)	1.81
Daytime Leisure	6	24	0.44	−7.34	5.45	+1.04 (32.81%)	1.17
Night-Time Leisure	9	6	0.32	−8.25	1.54	+1.17 (35.45%)	0.97
Aggregated data	70	119	0.55	−5.72	5.0	+1.47 (51.63%)	1.7

**Table 2:** Mean daily noise indices during the lockdown (2020) and the same time-frame for previous years (2018–2019) for the sensors in Heavy-Traffic Areas.

Period	$L_{d}$ [dB]	$L_{e}$ [dB]	$L_{n}$ [dB]
2018–2019	70.86	69.84	64.97
2020	66.63	64.21	58.11

**Table 3:** Subjective acoustic satisfaction assessment before and during the lockdown for dwellings near sensors in Heavy-Traffic Areas (Likert Scale).

Period	Very Negative	Negative	Neutral	Positive	Very Positive
Pre-Lockdown	9.09%	40.91%	13.64%	31.82%	4.55%
Lockdown	0%	0%	4.55%	31.82%	63.64%

**Table 4:** Mean daily noise indices during the lockdown (2020) and the same time-frame for previous years (2018–2019) for the sensors in Moderate-Traffic Areas.

Period	$L_{d}$ [dB]	$L_{e}$ [dB]	$L_{n}$ [dB]
2018–2019	67.62	66.23	61.48
2020	62.56	59.77	54.53

**Table 5:** Subjective acoustic satisfaction assessment before and during the lockdown for dwellings near sensors in Moderate-Traffic Areas (Likert Scale).

Period	Very Negative	Negative	Neutral	Positive	Very Positive
Pre-Lockdown	35.29%	17.65%	29.41%	17.65%	0%
Lockdown	0%	0%	5.88%	47.06%	47.06%

**Table 6:** Mean daily noise indices during the lockdown (2020) and the same time-frame for previous years (2018–2019) for the sensors in Low-Traffic Areas.

Period	$L_{d}$ [dB]	$L_{e}$ [dB]	$L_{n}$ [dB]
2018–2019	62.18	60.26	54.35
2020	57.86	54.08	49

**Table 7:** Subjective acoustic satisfaction assessment before and during the lockdown for dwellings near sensors in Low-Traffic Areas (Likert Scale).

Period	Very Negative	Negative	Neutral	Positive	Very Positive
Pre-Lockdown	34.62%	7.69%	34.62%	11.54%	11.54%
Lockdown	7.69%	3.85%	3.85%	42.31%	42.31%

**Table 8:** Mean daily noise indices during the lockdown (2020) and the same time-frame for previous years (2018–2019) for the sensors in SuperBlocks areas.

Period	$L_{d}$ [dB]	$L_{e}$ [dB]	$L_{n}$ [dB]
2018–2019	68.91	66.24	62.23
2020	61.69	58.91	55.19

**Table 9:** Subjective acoustic satisfaction assessment before and during the lockdown for dwellings near sensors in SuperBlocks areas (Likert Scale).

Period	Very Negative	Negative	Neutral	Positive	Very Positive
Pre-Lockdown	0%	28.57%	28.57%	28.57%	14.29%
Lockdown	0%	14.29%	0%	14.29%	71.43%

**Table 10:** Mean daily noise indices during the lockdown (2020) and the same time-frame for previous years (2018–2019) for the sensors in Day-time Leisure Areas.

Period	$L_{d}$ [dB]	$L_{e}$ [dB]	$L_{n}$ [dB]
2018–2019	63.72	64.3	57.03
2020	57.28	54.02	47.35

**Table 11:** Subjective acoustic satisfaction assessment before and during the lockdown for dwellings near sensors in Day-time Leisure Areas (Likert Scale).

Period	Very Negative	Negative	Neutral	Positive	Very Positive
Pre-Lockdown	8.33%	4.17%	58.33%	20.83%	8.33%
Lockdown	0%	0%	12.5%	54.17%	33.33%

**Table 12:** Mean daily noise indices during the lockdown (2020) and the same time-frame for previous years (2018–2019) for the sensors in Night-Time Leisure Areas.

Period	$L_{d}$ [dB]	$L_{e}$ [dB]	$L_{n}$ [dB]
2018–2019	63.97	64.54	62.74
2020	57.6	54.81	49.49

**Table 13:** Subjective acoustic satisfaction assessment before and during the lockdown for dwellings near sensors in Night-Time Leisure Areas (Likert Scale).

Period	Very Negative	Negative	Neutral	Positive	Very Positive
Pre-Lockdown	0%	0%	66.67%	33.33%	0%
Lockdown	0%	0%	0%	50%	50%

## Data Availability

No new data were created or analyzed in this study. Data sharing is not applicable to this article.

## Abbreviations

The following abbreviations are used in this manuscript:

ANR	Active Noise Reduction
EU	European Union
$L_{Aeq}$	A-weighted equivalent sound level
$L_{d}$	Day Noise Level. A-weighted $L_{eq}$, over the 7 h to 21 h period
$L_{e}$	Evening Noise Level. A-weighted $L_{eq}$, over the 21 h to 23 h period
$L_{n}$	Night Noise Level. A-weighted $L_{eq}$, over the 23 h to 7 h period
$L_{den}$	Day-Evening-Night noise level. A-weighted $L_{eq}$, over a whole day, but with a penalty of 10 dBA for night-time noise (23 to 7) and 5 dBA for evening noise (21 to 23)
SPL	Sound Pressure Level
WASN	Wireless Acoustic Sensor Network
WHO	World Health Organization
